# Willingness to attend cardiopulmonary resuscitation training and the associated factors among adults in China

**DOI:** 10.1186/s13054-020-03165-1

**Published:** 2020-07-23

**Authors:** Shijiao Yan, Yong Gan, Rixing Wang, Xingyue Song, Ning Zhou, Chuanzhu Lv

**Affiliations:** 1 School of Public Health, Hainan Medical University, Haikou, Hainan China; 2 Key Laboratory of Emergency and Trauma of Ministry of Education, Hainan Medical University, Haikou, Hainan China; 3 Department of Social Medicine and Health Management, School of Public Health, Tongji Medical College, Huazhong University of Science and Technology, Wuhan, Hubei China; 4 Department of Emergency, Hainan Clinical Research Center for Acute and Critical Diseases, The Second Affiliated Hospital of Hainan Medical University, No. 3 Xueyuan Road, Longhua Zone, Haikou, 571199 Hainan China; 5 Central People’s Hospital of Zhanjiang, No. 2 Cunjin Road, Chikan District, Zhanjiang, 524037 Guangdong China; 6 Emergency and Trauma College, Hainan Medical University, Haikou, Hainan China; 7 Research Unit of Island Emergency Medicine, Chinese Academy of Medical Sciences (No. 2019RU013), Hainan Medical University, Haikou, Hainan China

**Keywords:** Out-of-hospital cardiac arrest, Resuscitation, Cardiopulmonary resuscitation, Willingness, Training, China

Out-of-hospital cardiac arrest (OHCA) is an important public health challenge worldwide [1]. The survival rate of OHCA is less than 1% in China compared with 12% in the USA [2]. Previous studies have shown that immediate bystander-initiated cardiopulmonary resuscitation (CPR) and early defibrillation are essential to improve survival after OHCA [3]. However, the bystander CPR and CPR training rates remain insufficient, even in Western countries. This observational, national, cross-sectional survey aimed to investigate the prevalence of previous CPR training and willingness to be trained in CPR in the Chinese adults and to determine the associated factors.

A cross-sectional study was carried out in China from December 2018 to February 2019. A multistage stratified random sampling design was used in this study. First, a total of 31 Chinese provinces were classified as developed, developing, and less-developed regions according to per capita household income in 2018. Second, we selected 10 urban communities and 10 rural towns randomly from each province. Third, according to the number of residents and the scale of the community or town, from each sampled community or town, 30% of the residents who had lived in that county (or district) for at least 6 months were randomly selected to complete a self-administered questionnaire. Figure 1 shows the flowchart for recruitment and response rates. The questionnaire was shown in Supplement.

**Fig. 1:**
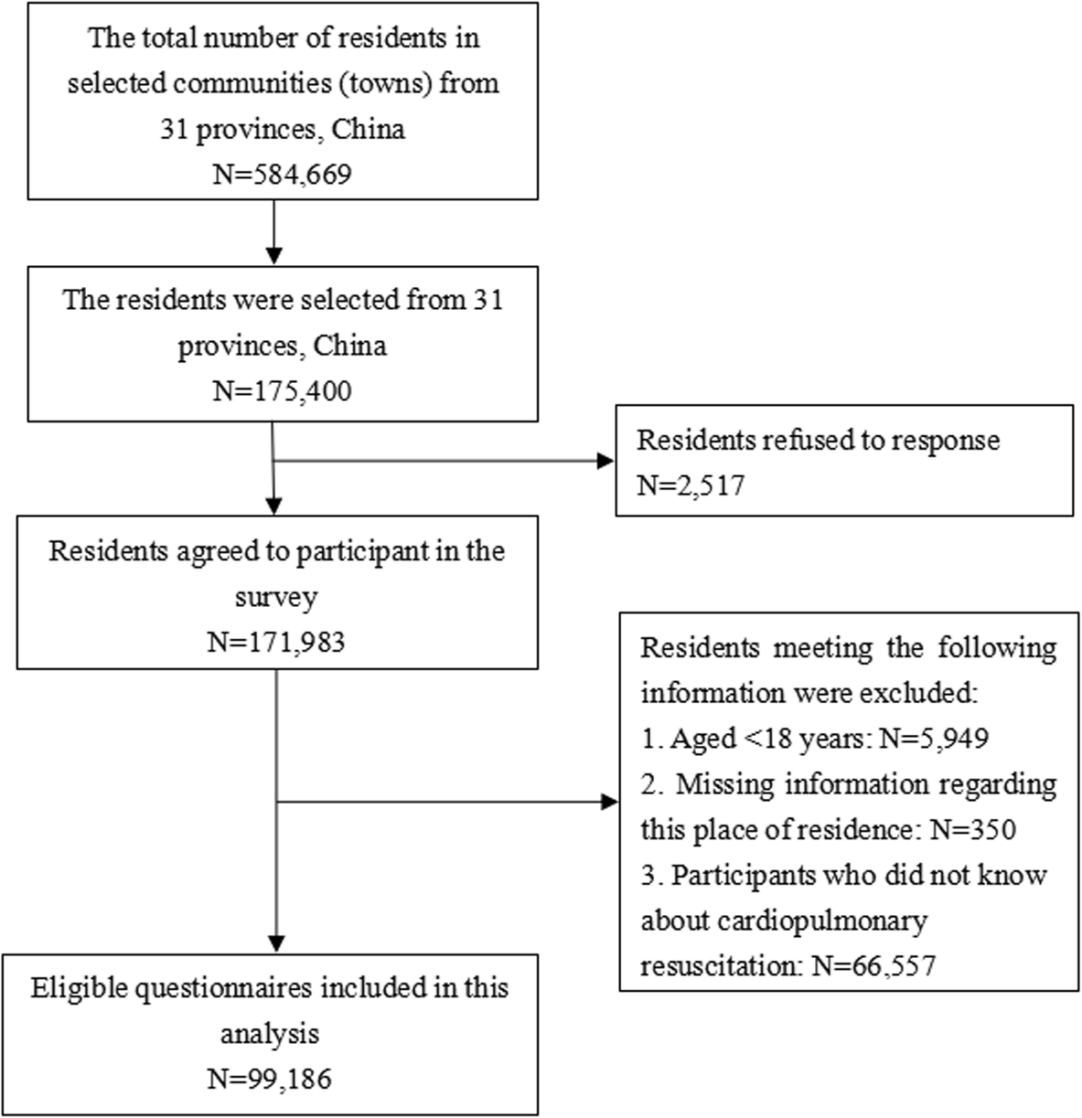
Flowchart for recruitment and response to the survey

Among the 99,186 respondents, more than half were women, and 59.9% were from developed regions. A minority of participants (37.6%) (*n* = 37,278) had attended a previous CPR training, and 21.5% respondents (*n* = 21,299) were familiar with automated external defibrillator (AED).

Overall, 73.4% of participants reported that they were willing to attend CPR training, 9.0% were unwilling to attend CPR training, and 17.7% were unsure of their willingness.

Participants who were female (OR = 1.68), had a higher education level (senior high school: OR = 1.47; college degree: OR = 1.77; bachelor’s degree or above: OR = 2.05), and had lower income (middle: OR = 1.62; low: OR = 1.61) were more likely to be willing to learn CPR. In addition, Chinese adults who had prior CPR training (OR = 1.70) and who were familiar with utilizing an AED (OR = 1.15) had high odds of attending CPR training. (Table 1).

**Table 1:** Logistic stepwise regression analysis for the associationwith the willingness to attend CPR training among adults

Variable	***Estimate***	***SE***	***Wald***	***P***	***OR*** (91% CI)
Gender (ref. male)					
Female	0.52	0.02	991.34	< 0.001	1.67 (1.62–1.73)
Age (ref. 60~)			48.71	< 0.001	
45~59	0.32	0.05	46.06	< 0.001	1.37 (1.25–1.50)
18~44	0.31	0.05	44.96	< 0.001	1.37 (1.25–1.50)
Ethnicity (ref. Han ethnicity)					
Minority	0.08	0.03	8.95	0.003	1.08 (1.03–1.14)
Education level (ref. junior high school and below)			763.31	< 0.001	
Senior school	0.39	0.03	156.32	< 0.001	1.47 (1.39–1.57)
College degree	0.57	0.03	359.77	< 0.001	1.77 (1.67–1.88)
Bachelor degree or above	0.72	0.03	701.69	< 0.001	2.05 (1.94–2.16)
Marital status (ref. unmarried/widow/divorced)					
Married	0.22	0.02	108.84	< 0.001	1.25 (1.19–1.30)
Work status (ref. retire)			76.97	< 0.001	
Unemployment	0.21	0.05	19.45	< 0.001	1.23 (1.12–1.35)
Employment	0.31	0.05	48.08	< 0.001	1.37 (1.25–1.50)
Health insurance (ref. no)					
Yes	0.33	0.03	144.59	< 0.001	1.40 (1.32–1.47)
Income status (ref. high)			637.90	< 0.001	
Middle	0.48	0.02	614.25	< 0.001	1.62 (1.56–1.68)
Low	0.48	0.02	365.77	< 0.001	1.61 (1.53–1.69)
Self-perceived health status (ref. poor)			70.81	< 0.001	
Fair	0.32	0.04	70.76	< 0.001	1.37 (1.27–1.48)
Good	0.28	0.04	55.39	< 0.001	1.32 (1.23–1.42)
Cigarette smoking (ref. smokers)	0.37	0.02	348.21	< 0.001	1.45 (1.39–1.51)
Non-smokers					
Physical inactivity (ref. yes)	0.26	0.02	272.18	< 0.001	1.29 (1.25–1.33)
No					
History of chronic disease (ref. yes)	0.15	0.02	45.44	< 0.001	1.16 (1.11–1.21)
No					
Having previous CPR training (ref. no)	0.53	0.02	934.69	< 0.001	1.70 (1.64–1.76)
Yes					
Familiarity with utilizing an AED (ref. no)	0.14	0.02	47.17	< 0.001	1.15 (1.11–1.20)
Yes					
Constant	− 2.21	0.07	969.67	< 0.001	0.11

*Abbreviations*: *AED* automated external defibrillator, *CPR* cardiopulmonary resuscitation

The percentage of adults with CPR training was higher than that reported for the general population in Japan (35%) [4], but lower than in most developed countries, such as Sweden (45%) [5] and Crimea (53%) [6]. The differences might be due, at least in part, to differences in sample size and the participants’ characteristics, including their socioeconomic status and the CPR training awareness in their national context.

Our study showed that 73.4% of the respondents were willing to learn CPR, which was higher than in previous studies conducted in other countries [6]. These findings indicate that CPR training is highly acceptable to the public. Previous research has shown that socioeconomic disparities exist with regard to attending CPR training and surviving an OHCA [6]. The findings suggest that willingness to attend CPR training may also correlate with socioeconomic factors, specifically with educational attainment. We identified an independent association between having a higher education level and an increased likelihood of attending CPR training.

No significant association between place of residence and willingness of attend CPR training was found in our study, which was consistent with a previous study [6]. However, a previous study conducted by Axelsson et al. [5] showed that urban residents were more willing to learn CPR. This finding may signal to institutions to provide equal CPR training opportunities and projects for rural and urban residents in China.

The Chinese government should make efforts to optimize and standardize a national model of CPR delivery training and enhance the public awareness and motivation to increase the willingness to attending CPR training.

## Supplementary information

**Additional file 1.**

## Data Availability

Data may be made available by contacting the corresponding author.

## Abbreviations

AED: Automated external defibrillator
CI: Confidence interval
CPR: Cardiopulmonary resuscitation
OHCA: Out-of-hospital cardiac arrests
OR: Odds ratio

## References

[1] Berdowski J, Berg RA, Tijssen JG, Koster RW (2010). Global incidences of out-of-hospital cardiac arrest and survival rates: systematic review of 67 prospective studies. Resuscitation.

[2] Hua W, Zhang LF, Wu YF, Liu XQ, Guo DS, Zhou HL, Gou ZP, Zhao LC, Niu HX, Chen KP (2009). Incidence of sudden cardiac death in China: analysis of 4 regional populations. J Am Coll Cardiol.

[3] Malta Hansen C, Kragholm K, Pearson DA, Tyson C, Monk L, Myers B, Nelson D, Dupre ME, Fosbol EL, Jollis JG (2015). Association of bystander and first-responder intervention with survival after out-of-hospital cardiac arrest in North Carolina, 2010-2013. JAMA.

[4] Kuramoto N, Morimoto T, Kubota Y, Maeda Y, Seki S, Takada K, Hiraide A (2008). Public perception of and willingness to perform bystander CPR in Japan. Resuscitation.

[5] Axelsson AB, Herlitz J, Holmberg S, Thoren AB (2006). A nationwide survey of CPR training in Sweden: foreign born and unemployed are not reached by training programmes. Resuscitation.

[6] Birkun A, Kosova Y (2018). Social attitude and willingness to attend cardiopulmonary resuscitation training and perform resuscitation in the Crimea. World J Emerg Med.

