# Supplementary material for: Willingness to attend cardiopulmonary resuscitation training and the associated factors among adults in China
Source: Crit Care. 2020 Jul 23;24:457. doi: 10.1186/s13054-020-03165-1 (PMC7376923; doi:10.1186/s13054-020-03165-1)
Supplement: Supplementary file 1 — Additional file 1. [file 13054_2020_3165_MOESM1_ESM.docx]

**公众急救知识和健康状况调查问卷**

尊敬的朋友：

您好！我们是海南医学院院前急救课题组。为了解我国公众急救知识及健康状况，提

高公众的现场急救能力和促进人群健康，特开展本次调查。本调查采用匿名方式填写，调查结果仅用作统计分析，我们将对数据进行严格保密，感谢您的配合！祝万事如意！

海南医学院

华中科技大学公共卫生学院

**一、以下问题主要了解您的基本信息，请根据自己实际情况填写或选择相应信息。**

1. 您的年龄_______岁 [填空题] *

_________________________________

2. 性别： [单选题] *

| ○男 |
| --- |
| ○女 |

3. 您的居住地是： [填空题] *

_________________________________

4. 您目前长期居住的地方为： [单选题] *

| ○城市 |
| --- |
| ○农村 |

5. 目前居住地是否为户口所在地： [单选题] *

| ○是 |
| --- |
| ○否 |

6. 民族： [单选题] *

| ○汉族 |
| --- |
| ○少数民族 _________________ |

7. 文化程度： [单选题] *

| ○小学及以下 |
| --- |
| ○初中 |
| ○高中或中专 |
| ○大专 |
| ○本科 |
| ○硕士及以上 |

8. 婚姻状况： [单选题] *

| ○未婚 |
| --- |
| ○已婚 |
| ○离异 |
| ○丧偶 |

9. 工作类型： [单选题] *

| ○无工作 |
| --- |
| ○正式全职工作 |
| ○兼职工作 |
| ○离/退休 |

10. 您参加的医疗保险类型： [单选题] *

| ○城镇职工医疗保险 |
| --- |
| ○城乡居民基本医疗保险(包括新农合) |
| ○商业保险 |
| ○公费医疗 |
| ○没有任何保险 |

11. 您认为，您目前的经济状况如何？ [单选题] *

| ○很好 |
| --- |
| ○比较好 |
| ○一般 |
| ○比较差 |
| ○很差 |

12. 您认为，您目前的健康状况如何？ [单选题] *

| ○很好 |
| --- |
| ○比较好 |
| ○一般 |
| ○比较差 |
| ○很差 |

13. 最近半年，您平均每天抽烟的支数： [单选题] *

| ○不抽烟 |
| --- |
| ○少于1支 |
| ○1-5支 |
| ○6-10支 |
| ○11-20支 |
| ○＞20支 |

14. 最近半年，您平均每周喝酒的次数： [单选题] *

| ○不饮酒 |
| --- |
| ○少于1次 |
| ○1-3次 |
| ○4-7次 |
| ○8-14次 |
| ○＞14次 |

15. 您目前运动频率（需每次坚持30分钟以上才算运动，包括散步、瑜伽等） [单选题] *

| ○不运动或很少运动 |
| --- |
| ○每周1-2次 |
| ○每周3-5次 |
| ○每周6-7次 |

16. 目前，您是否患有慢性疾病(经医生确诊的)？ [单选题] *

| ○无 |
| --- |
| ○有 |

**二、以下问题主要想了解一下您对急救知识的了解、培训等情况，请选择符合您实际情况的选项。**

17. 您是否知道什么是心肺复苏？ [单选题] *

| ○是 |
| --- |
| ○否 **(请跳至第37题)** |

18. 您了解心肺复苏的主要途径是？ [多选题] ***可多选**

| □网络 |
| --- |
| □电视、广播 |
| □报纸、杂志、书本 |
| □专业培训、健康教育讲座 |
| □其他 _________________ |

19. 您是否参加过心肺复苏的培训？ [单选题] *

| ○是 |
| --- |
| ○否 |

20. 您愿意参加心肺复苏的培训吗？ [单选题] *

| ○非常不愿意 |
| --- |
| ○不愿意 |
| ○不确定 |
| ○愿意 |
| ○非常愿意 |

21. 您认为该如何判断成年患者有无意识？ [单选题] *

| ○大声呼唤、轻拍肩膀 |
| --- |
| ○大声呼唤、摇晃肩膀 |
| ○大声呼唤、拍面颊 |
| ○不知道 |

22. 若确定患者失去意识，无呼吸心跳，应尽力在“黄金时间”_________ 分钟内进行心肺复苏？ [单选题] *

| ○4分钟内 |
| --- |
| ○10分钟内 |
| ○30分钟内 |
| ○不知道 |

23. 进行心肺复苏时应首先实施 _________？ [单选题] *

| ○开放气道 |
| --- |
| ○人工呼吸 |
| ○胸外按压 |
| ○不知道 |

24. 胸外心脏按压时患者适宜躺在_________？ [单选题] *

| ○柔软床上 |
| --- |
| ○沙发上 |
| ○硬板床或硬地板上 |
| ○不知道 |

25. 心跳呼吸骤停的患者应安置其何种体位？ [单选题] *

| ○侧卧位 |
| --- |
| ○仰卧位 |
| ○俯卧位 |
| ○不知道 |

26. 对成年患者进行胸外按压的部位是？ [单选题] *

| ○胸部左侧 |
| --- |
| ○胸部中央 |
| ○胸部右侧 |
| ○不知道 |

27. 对成年患者进行胸外心脏按压时手的姿势为？ [单选题] *

| ○双手重叠五指按压 |
| --- |
| ○两只手掌根重叠按压，手指贴紧胸壁 |
| ○两只手掌根重叠按压，手指离开胸壁 |
| ○不知道 |

28. 对成年患者进行胸外心脏按压时，手臂的姿势为？ [单选题] *

| ○两只手臂姿势随意 |
| --- |
| ○两只手臂弯曲 |
| ○两只手臂伸直，且垂直于患者胸部 |
| ○不知道 |

29. 对成年患者进行胸外按压时，按压的深度为？ [单选题] *

| ○3-4厘米 |
| --- |
| ○至少5厘米 |
| ○5-6厘米 |
| ○不知道 |

30. 对成年患者进行胸外按压时，每分钟按压的次数？ [单选题] *

| ○80次/分 |
| --- |
| ○至少100次/分 |
| ○100-120次/分 |
| ○不知道 |

31. 当进行口对口人工呼吸时，被救者头部应如何放置？ [单选题] *

| ○头部平仰 |
| --- |
| ○下颌抵住胸 |
| ○头部后仰、抬高下颌 |
| ○不知道 |

32. 怎样进行口对口人工呼吸吹气？ [单选题] *

| ○捏鼻子，包住口 |
| --- |
| ○捏鼻子，不包住口 |
| ○不捏鼻子，包住口 |
| ○不知道 |

33. 当进行心肺复苏时，心脏按压与人工呼吸的次数应按怎样比例交替进行？ [单选题] *

| ○20:2 |
| --- |
| ○30:2 |
| ○40:2 |
| ○不知道 |

34. 您知道什么是除颤器吗？ [单选题] *

| ○知道 |
| --- |
| ○不知道 |

35. 您知道公共场所除颤器的标志吗？ [单选题] *

| ○知道 |
| --- |
| ○不知道 |

36. 您知道如何使用除颤器吗？ [单选题] *

| ○知道 |
| --- |
| ○不知道 |

37. 中国的首都在哪里？ [单选题] *

| ○北京 |
| --- |
| ○重庆 |
| ○天津 |
| ○杭州 |

**三、以下问题主要想了解您在过去的两周时间内出现相应症状的频率，请选择符合您实际情况的选项。**

38. 做什么事都没兴趣、沒意思 [单选题] *

| ○没有 |
| --- |
| ○有几天 |
| ○一半以上时间 |
| ○几乎天天 |

39. 感到心情低落、抑郁、沒希望 [单选题] *

| ○没有 |
| --- |
| ○有几天 |
| ○一半以上时间 |
| ○几乎天天 |

40. 入睡困难、总是醒着、或睡得太多嗜睡 [单选题] *

| ○没有 |
| --- |
| ○有几天 |
| ○一半以上时间 |
| ○几乎天天 |

41. 常感到很疲倦、沒劲 [单选题] *

| ○没有 |
| --- |
| ○有几天 |
| ○一半以上时间 |
| ○几乎天天 |

42. 口味不好，或吃的太多 [单选题] *

| ○没有 |
| --- |
| ○有几天 |
| ○一半以上时间 |
| ○几乎天天 |

43. 自己对自己不满, 觉得自己是个失败者,或让家人丟脸了 [单选题] *

| ○没有 |
| --- |
| ○有几天 |
| ○一半以上时间 |
| ○几乎天天 |

44. 无法集中精力,即便是读报纸或看电视时,记忆力下降 [单选题] *

| ○没有 |
| --- |
| ○有几天 |
| ○一半以上时间 |
| ○几乎天天 |

45. 行动或说话缓慢到引起人们的注意，或刚好相反，坐卧不安,烦躁易怒易怒,到处走动 [单选题] *

| ○没有 |
| --- |
| ○有几天 |
| ○一半以上时间 |
| ○几乎天天 |

46. 有不如一死了之的念头, 或想怎样伤害自己一下 [单选题] *

| ○没有 |
| --- |
| ○有几天 |
| ○一半以上时间 |
| ○几乎天天 |

调查日期：2018年____月____日
